# Supplementary material for: Evaluation of a modified quantitative polymerase chain reaction assay for genus Schistosoma detection using stool and urine samples from schistosomiasis endemic areas in Kenya
Source: PLoS One. 2024 Sep 20;19(9):e0310118. doi: 10.1371/journal.pone.0310118 (PMC11414982; doi:10.1371/journal.pone.0310118)
Supplement: S1 Appendix — (DOCX) [file pone.0310118.s001.docx]

**S1 Appendix: World Health Organization (WHO) intensity thresholds for light, moderate and heavy infections for *Schistosoma***

| **Helminth*** | **Intensity Threshold** | | |
| --- | --- | --- | --- |
|  | **Light** | **Moderate** | **Heavy** |
| *S. mansoni* | 1 – 99 epg | 100 – 399 epg | ≥ 400 epg |
| *S. haematobium* | 1 – 50 eggs/10ml urine | - | ≥ 50 eggs/10ml urine |

*These intensity thresholds were adopted from a WHO report of 2002 on helminth control in school age children [28,29]
