# Supplementary material for: Evaluation of a modified quantitative polymerase chain reaction assay for genus Schistosoma detection using stool and urine samples from schistosomiasis endemic areas in Kenya
Source: PLoS One. 2024 Sep 20;19(9):e0310118. doi: 10.1371/journal.pone.0310118 (PMC11414982; doi:10.1371/journal.pone.0310118)
Supplement: S2 Appendix — (DOCX) [file pone.0310118.s002.docx]

**Supplementary Appendix 2 (S2 Appendix)**

**Bayesian Latent Class Model**

The Bayesian Latent Class Model (BLCM) was used for the data analysis.

It is assumed that the test results are distributed according to a multinomial model for the observed counts (O_k_) in each population as shown below:

O_k_ | Se_ik_Sp_ik_P_k_ ~ multinomial (prob_k_,n_k_)

Where Se_ik_ and Sp_ik_ are the sensitivity and specificity measures for the i^th^ test (i=1,2) in the given sub-population represented by k in the equation (k=1,2) and P_k_ represent the k^th^ subpopulation’s prevalence. Prob_k_ is a vector of probabilities of observing the different combination of tests results while n_k_ is the sample size used in sub-population k. The probabilities are defined using the specific test characteristics that is sensitivity and specificity and prevalence for each sub-population. For example, in the first population for individuals testing positive to both tests, prob_1_ is given by:

Prob_1_= Pr (T_1_^+^ T_2_^+^ | D^+^) + Pr (T_1_^+^ T_2_^+^ | D^-^) = Se_11_ Se_21_ P_1_ + [1-Sp_11_] [1-Sp_21_] [1-P]

Where T_1_^+^ denotes an individual that tests positive in test 1; T_2_^+^ denotes an individual that tests positive in test 2; D^+^ denotes a diseased individual; D ^-^ denotes a non- diseased individual.

The diagnostic performance of the tests was further assessed in terms of positive predictive value (PPV) and negative predictive value (NPV). Therefore, the PPV and NPV for the i ^th^ test and sub-population k was calculated as follows:

PPV= P_k_ Se_ik_ / (P_k_ Se_ik_ + [ 1- P_k]_ [1- Sp_ik_])

NPV= [ 1- P_k]_ Sp_ik_ / (P_k_ [1- Se_ik]_ + / (P_k_ Se_ik_ + [ 1- P_k]_ [1- Sp_ik_])

The model was initialized by two Markov Chain Monte Carlo chains (MCMC). Each chain was iterated 100,000 times with the initial 50,000 runs discarded as burn-in. The convergence of the chains was visually assessed through the time-series plots of selected variables and Gelman- Rubin diagnostic plots. The test estimates of sensitivity and specificity and the posterior distribution of the prevalence of the sub-populations were reported as the median value with the associated posterior confidence interval (PCI).
