# Supplementary material for: Evaluation of a modified quantitative polymerase chain reaction assay for genus Schistosoma detection using stool and urine samples from schistosomiasis endemic areas in Kenya
Source: PLoS One. 2024 Sep 20;19(9):e0310118. doi: 10.1371/journal.pone.0310118 (PMC11414982; doi:10.1371/journal.pone.0310118)
Supplement: Attachment — Submitted filename: Response to Reviewers.docx [file pone.0310118.s004.docx]

Racquel Inocencio da Luz, PhD

Academic Editor

PLOS ONE

30^th^ July 2024

Dear Dr. Racquel Inocencio da Luz,

**Re: Revised version of manuscript reference PONE-D-24-12369**

Thank you for giving us an opportunity to revise our manuscript entitled " Evaluation of a modified quantitative polymerase chain reaction assay for genus *Schistosoma* detection using stool and urine samples from schistosomiasis endemic areas in Kenya " for submission to PLOS ONE. We are grateful for the valuable feedback from both you and the reviewers, which has helped us to enhance the paper. We are pleased to resubmit the article for your further consideration, incorporating changes based on your insightful suggestions. We believe our revisions and the responses provided below adequately address the issues and concerns raised

For your review here is a point by point response to the comments and questions raised. The comments are indicated in bold while our responses are not bold.

**Journal Requirements**

**1. Please ensure that your manuscript meets PLOS ONE's style requirements, including those for file naming.**

The manuscript has been reformatted as per the requirement of PLOS ONE’s requirements

**2. Funding Information and Financial Disclosure sections do not match: ""We note that the grant information you provided in the ‘Funding Information’ and ‘Financial Disclosure’ sections do not match. When you resubmit, please ensure that you provide the correct grant numbers for the awards you received for your study in the ‘Funding Information’ section.**

The text in the “Funding Information” and “Financial Disclosure” sections have been rewritten to match as follows:

“This work was supported by the Bill and Melinda Gates Foundation (grant award: INV_024908) that was awarded to SH. The funder had no role in decision to publish, or preparation of the manuscript.”

**3. In the online submission form, you indicated that "All relevant data are within the manuscript and its Supporting Information files. The raw data will be available upon request"**

**All PLOS journals now require all data underlying the findings described in their manuscript to be freely available to other researchers, either 1. In a public repository, 2. Within the manuscript itself, or 3. Uploaded as supplementary information.
This policy applies to all data except where public deposition would breach compliance with the protocol approved by your research ethics board. If your data cannot be made publicly available for ethical or legal reasons (e.g., public availability would compromise patient privacy), please explain your reasons on resubmission and your exemption request will be escalated for approval.**

Kindly note that the consent forms utilized to collect data from participants explicitly stated that access to the data is limited to study staff, representatives from FIND and KEMRI, members of the ethics committee and the regulatory authorities. Therefore, access to anonymized and de-identified data can be obtained by emailing the Kenya Medical Research Institute (KEMRI) Scientific Ethics Review Unit (SERU) at [seru@kemri.go.ke](mailto:seru@kemri.go.keom)

**4. Please ensure that you refer to Figure 1 in your text as, if accepted, production will need this reference to link the reader to the figure.**

Reference to Figure 1 has been provided in lines 159 – 161. The figure has been updated. The source has also been acknowledged and the access date provided accordingly.

**5. Please include captions for your Supporting Information files at the end of your manuscript, and update any in-text citations to match accordingly.**

Thank you for this comment we have provided captions at the end of the manuscript for the supporting information and have updated in- text citations to match accordingly (Line 677 - 680) & (Line 184 - 193 ;Line 248 - line 281)

**6. Please review your reference list to ensure that it is complete and correct. If you have cited papers that have been retracted, please include the rationale for doing so in the manuscript text, or remove these references and replace them with relevant current references. Any changes to the reference list should be mentioned in the rebuttal letter that accompanies your revised manuscript. If you need to cite a retracted article, indicate the article’s retracted status in the References list and also include a citation and full reference for the retraction notice.**

Based on the recommendations from the reviewers some other references have been introduced and captured accordingly.

**Reviewer 1 Comments**

**1. It would be important for the authors to mention the chemotherapeutic strategies that have been deployed for schistosomiasis control and any successes that have been achieved using this strategy in the introduction section of the manuscript**

Agreed, this has been added in the introduction section. A paragraph in the introduction section has been added to give context to the main chemotherapeutic strategy used for schistosomiasis through mass drug administration (MDA) of praziquantel. This strategy has mostly been implemented by the National School Based Deworming Programme (NSBDP) and has shown some impact on lowered the overall prevalence of schistosomiasis after repeated rounds of MDAs. The paragraph is on Line 64 - 70.

**2. In lines 86–92, where the authors highlight the limitations of current diagnostic tests, the requirement for well-trained and experienced personnel is mentioned. I am wondering if the qPCR test developed in this manuscript would solve this problem. In addition, how suitable is the developed test for low-resource settings? In the same light, the authors mention qPCR as being advantageous over conventional PCR (lines 117–119), but what about the sophistication and equipment?**

We thank Reviewer 1 for the above comments. Please note that our recommendations are as follows (Line 468 – 471):- It is therefore recommended for use by the national programmes for accurate determination elimination endpoints and disease surveillance to avert the re-emergence of the infection after effective control. However, to mitigate the cost, a subset of the sample size could be analyzed using the assay as a proxy indicator of the infection level.

We are cognizant that cost is a limiting factor. However, the recommended test is not for routine use but rather for determination of the diseases elimination endpoints. Further, we recommend that a subset can be selected based on the available funding. This will enable striking of the balance between cost and accurate determination of the disease elimination.

**3. Concerning the ethical considerations, the authors mention that participants provided written informed consent. Here are my concerns about this: Were all participants above the age of consent? Were all participants literate? How was consent obtained from the illiterate and minors? More clarity is needed on this.**

The points raised have valid concerns. We have added the following information in the ethics statement to clarify these matters. For participants less than 18 years old, parental consent was obtained, with assent from children (13- <18 years) before enrollment. Illiterate participants provided a thumbprint on the consent form and the consent form was also signed by an impartial witness. This information is provided in Line 133 - 136.

**4. Concerning the study sites selected, the authors should provide any previous data on *Schistosoma* prevalence. In addition, the authors should provide consistent data on the different study sites (e.g., rainfall, economic activity, humidity, temperature, etc)**

In response to the comments above we have done the following:-

i. The three study sites have been amended to provide consistent information on the general location, economic activities, general climatic conditions and rainfall. Line 140 - 157
ii. We have provided the data supporting prevalences in the endemic sites and the reference for classification of the control site as non-endemic sites by the Ministry of Health, Kenya (Line 154)
iii. We have also provided a Kenyan map with updated endemicity for Schistosomiasis. In addition to the endemicity of the sites, the maps provide the location of the sites. Line 159 - 161

**5. Figure 1 should read, "Map of Kenya...", and not "Kenyan map...". In addition, I am wondering if permission is needed from ESPEN for this map to be used. In addition, I am wondering if there is no prevalence data that is more recent, given that this map was produced 6 years ago.**

The most recent map (2022) has been prepared, renamed and acknowledged as per review comments. Line 159 -161. With regard to permission to use the map (Figure 1), we reached out to ESPEN and advised that no permission is required, but only to acknowledge the source and date accessed.

**6. It would be important for the authors to provide information on other diagnostic parameters, like likelihood ratios, % positive agreement, Youden's index, etc).**

Thank you for this comment. As added in the discussion section they are two main methods of estimating sensitivity and specificity 1) method that utilizes a gold standard or reference test known as frequentists statistics 2) methods that do not utilize a gold standard known as Bayesian methods this is in Line 382 to 388. Bayesian statistics has been utilized in the data analysis of the paper. Cohen Kappa Index is a test of agreement that utilizes frequentist statistics. This also applies to likelihood ratios, % positive agreement but we have added Youden's index in table 4 of the manuscript in line 342 - 344

**7. In lines 435–437, the authors should provide specific sensitivity information on the study being referred to.**

The specific sensitivity of the study being referred to has been added that is the Tanzanian study found a sensitivity for Kato-Katz at 89.7% and sensitivity for qPCR at 98.7%. This is in line 428 - 430

**8. Lastly, the manuscript needs to be carefully proofread again and grammatical errors corrected. DNA (line 112) is an acid, the full meaning of NTD (line 146) should be provided. A consistent style should be used to indicate temperatures**

We thank Reviewer 1 for this comment. We have undertaken proofreading of the manuscript and corrected various grammatical errors throughout the manuscript.

**Reviewer 2 Comments**

1. **Abstract - Refer to the main text comments and apply the modifications here in to the abstract as well.**

We have made the necessary changes to the entire abstract

2. **Abstract Introduction- Move to methodology**

This sentence has been moved to methodology (Line 9 -11 moved to 16 -17)

3. **Abstract Methodology- Any particular reason why *S. japonicum* (being a species causing significant morbidity in SEA) was not looked in to?**

This is because *S. japonicum* is not geographically distributed in the African continent and is predominantly found in Asia. There was therefore no site to collect site from in Kenya for comparison

4. **Abstract Methodology - What types of samples?**

The type of samples has been added in brackets for clarity. Please see line 17 – 18.

5. **Abstract Results - Not clear how the denominator becomes 849, and not 699. This should focus exclusively on the endemic regions.**

Agreed and revised in line 21 -22. Please note that the study had 3 sites (two endemic and one non-endemic site) where samples were collected as follows: - In *S. mansoni* endemic site, 351 stool samples were collected while in *S. haematobium endemic* site 348 urine samples were collected. This added up to 699 samples in the two endemic sites. However, in non-endemic site, each of the 75 participants provided both stool and urine samples. Therefore, 150 samples (75 participants x 2 samples each) were collected in the non-endemic site. Overally then, 699 and 150 samples (total of 849) were collected.

6. **Abstract Results - Same as above**

Agreed, this has been edited as indicated above

7. **Abstract Results - Both microscopy and PCR is supposed to provide quantitative outcomes. A briefing of the comparison needs to come in here.**

Comparison has been provided on the performance of the two techniques (Line 25 to 27).

8. **Introduction - The diagnosis of schistosomiasis has been facilitated through the application of different PCR techniques, including conventional PCR, qPCR, as well as digital PCR (ddPCR). Suggest briefing with appropriate references.**

Thank you for this important suggestion; a topic sentence has been added at the beginning of the paragraph to introduce the concept of PCR techniques in control of schistosomiasis. This is found in line 97 to 99.

9. **Introduction- Specify the study objectives**

Agreed, this has been done on the last paragraph of the introduction (Line 121 to 124)

10. **Introduction- Comment on line 108 – 114 Shouldn’t be places in the Introduction, and instead can be incorporated in to the discussion. –**

Thank you for this insightful comment. However, although the statement can also fit in the discussion, I strongly feel that it also fits here for two reasons. The first reason is that it lays basis for the reader to understand the background/general scope of the work reported in this manuscript. Secondly, it exemplifies the how the current PCR method was different from other PCR done on the schistosomiasis and specifically why it is referred to as “modified” under Line 116 - 121

11. **Introduction- Not a abbreviation introduced above in the text**

Defined – line 118

12. **Materials and Methods- Study area- This section addresses both study type as well as the sites.**

Agreed, the title of this section has been amended to reflect the information found in this section. This is in Line 138

13. **Materials and Methods- Suggest citing the publication(s) arose from the primary study –** Line 139 - 140

Although the samples utilized in this study are from stored, there is no publication that has been done on these samples. Therefore it is expected that this will be the first publication and that will be cited by any subsequent publications if any.

14. **Materials and Methods- The figure appears to be from another publication/source. Need to cite the source with permissions.**

Line 159 - 161. With regard to permission to use the map (Figure 1), we reached out to ESPEN and advised that no permission is required, but only to acknowledge the source. Same response provided to similar comment by reviewer 1 in comment 5. Thanks.

15. **Materials and Methods- Not abbreviated previously**

Agreed, the text containing this abbreviation has been rephrased in line 167 – 170. The abbreviation was done in line 118

16. **Materials and Methods- Were these microscopic findings published previously? Please cite and link if done so.**

As indicated earlier the microscopy results are not yet published and thus this is first publication and which compares both the microscopy and PCR results. Line 173

17. **Materials and Methods- Either briefly describe or cite a reference to provide the methodological aspects.**

A citation is in place that contains the Kato-Katz technique used for stool examination (Reference Number 12) in line 187

18. **Materials and Methods- Outcome of two slides? How were the samples from three days considered (was it altogether six smears)? Specify**

Thank you for this comment, this has been clarified as follows:-. The samples were considered altogether from the six smears. A sample was considered to be positive if any of the slide was positive while a negative was where it was negative by all the slides. In the case of *S. mansoni,* eggs were counted and intensity expressed as eggs per gram of stool (epg) by multiplying the arithmetic mean of egg counts from the six slides per participant by 24 and categorized as per the WHO guidelines. In the case of *S. haematobium* the intensity of infection was calculated as the arithmetic mean of the eggs per 10 ml of urine from the six slides per participant and expressed as the numbers of eggs per 10 ml of urine as per the WHO guidelines, this can be found on Line 187 -193.

19. **Materials and Methods- Specify how the quality assurance was ensured in microscopic analysis**

Thank you for raising an important point. For quality assurance purposes, a random examination of 10% of the slides were re-examined by senior technologists. This is in Line 193 to 194

20. **Materials and Methods- Specify the ‘modification(s)’, showing how the modifications would have improved the detection sensitivity. The essence of the title also relates to this matter. Hence it is imperative to detail the related facts.**

The modification of the current PCR is indicated in Line 116 – 121. In addition in line 204 – 211, the current protocol also utilized a plasmid (pDMD801, 100pg/ul) as an internal control instead Phocin Herpes Virus 1 (PhHV-1) that was used in the previous protocol. This is described in the protocol too.

21. **Materials and Methods- What was the basis for this stratification. Were a series of DNA samples from microscopy-confirmed light, moderate, and heavy infections tested using qPCR, with corresponding cutoffs applied?**

The basis for this stratification was based on previous literature which has been cited by Obeng et al and Aryeetey et al which have been cited in this section. Line 198 and 217. However, the samples were not stratified by microscopy before PCR and which was a form of “Blinding”. This was done after PCR analysis was done

22. **Materials and Methods- What is the upper limit for a Ct of over 35? There should be an upper cutoff value beyond which the outcome is considered negative. Typically, amplifications beyond 40 are regarded as potentially nonspecific.**

Thank you for the comment. The cut off for PCR may differ from assay to assay depending with the specificity of the primers and the region of amplification subject to the optimization of the assay. For this assay, our positive is defined as a sample with any amplification and thus amplification above 35 is still positive and the higher cycles are attributable to low DNA concentration rather than nonspecific amplification. It for this reason that we define a negative as a sample whose CT = 0 (No amplification) – Line 241 - 242

23. **Materials and Methods- This does not fit in within the Methodology section. Instead, place this either in the Introduction, connected with the objectives, or incorporate this within the discussion."**

Agreed and the section deleted. Line 243 - 246

24. **Materials and Methods- After the first use, the genus name has been abbreviated to its first initial from the top. This need to be applied consistently and revise this throughout the text**

Thank you for this observation this has been done consistently throughout the manuscript.

25. **Materials and Methods- After the first use, the genus name has been abbreviated to its first initial from the top. This need to be applied consistently and revise this throughout the text.**

Thank you for this observation this has been done consistently throughout the manuscript.

26. **Materials and Methods- After the first use, the genus name has been abbreviated to its first initial from the top. This need to be applied consistently and revise this throughout the text.**

Thank you for this observation this has been done consistently throughout the manuscript.

27. **Results- The control group (non-endemic region) should not be taken in to consideration here. Specifically mention ‘The positive results by microscopy in the two endemic regions altogether was …..’ with the denominator as the total sample s from these two regions. It is understood that participants from the non-endemic region were recruited to serve as a control group**

Agreed, the control group (non- endemic region) should not be taken into consideration here. We have amended the text accordingly to read “The proportion of positive results by microscopy in the two endemic regions altogether was 289/699 (41.3%)” (Line 304 to 305)

28. **Results- This was expected.**

Agreed, this has been edited.

29. **Results- Suggest statistically testing the agreement between these parameters. E.g., with Cohen’s Kappa Index. This also applies to comparison between quantitative categories (table 2)**

Thank you for this comment. As added in the discussion section they are two main methods of estimating sensitivity and specificity 1) method that utilizes a gold standard or reference test known as frequentists statistics 2) methods that do not utilize a gold standard known as Bayesian methods this is in Line 382 to 388. Bayesian statistics has been utilized in the data analysis of the paper. Cohen Kappa Index is a test of agreement that utilizes frequentist statistics. This also applies to likelihood ratios, % positive agreement but we have added Youden's index in table 4 of the manuscript in line 342 – 344

30. **Results- Suggest tabulating BLCA analysis results and cite within the text.**

Thank you for this suggestion. We have added Table 4 (line 342) in the manuscript that shows the estimates of the sensitivity and specificity of PCR and microscopy and their respective 95 % posterior credibility interval (PCI) for diagnosis of *Schistosoma* per site which were obtained using the Bayesian Latent Class Model analysis (BLCM)

31. **Results on higher sensitivity of Microscopy in *S. haematobium* - This need to be discussed**

Done Line 414 – 418

32. **Discussion- Discuss how this model would be better than conventional Se Sp calculations.**

Thank you for this comment. The information has been added in the discussion section of the paper. Traditionally, methods of estimating sensitivity and specificity are based on the premise that there is a gold standard or reference tests [50]. A drawback of this approach is that, given the imperfection of these reference tests the results generated may be prone to misclassification errors. On the other hand, BCLM allows estimation of the sensitivity and specificity without assumptions of the true disease status of each individual [49,51]. Additionally, the model allows incorporation of prior information from literature about parameters such as sensitivity, specificity or prevalence enhancing the estimates generated this is in line 382 to 388

33. **Discussion - As previously queried (Lines 335-336), it is important to discuss the fact that Se from microscopy was better than Se of PCR in *S. haematobium* infection. How can this be explained?**

Discussed in line 414 – 418

It is worth noting that urine filtration and urine PCR utilized 10mls and 1ml of neat urine samples respectively. Conversely, Kato katz utilized 41.7g of stool while stool PCR utilized sample preserved in absolute ethanol at 1:3 mass to volume ratio. This could probably have effects on the recovery of eggs and detection of the infection in favour of *S. haematobium* microscopy due to samples volumes compared its PCR and both microscopy and PCR in *S. mansoni*.

34. **Discussion- How could this be explained. Also discuss / explain the occurrence of the other way around as well**

This is not totally unexpected because of the documented superior sensitivity by PCR over Microscopy. Thus, PCR could easily pick a single egg that is missed by Microscopy. This could probably also explain why there was increase in numbers of individuals with heavy infection by the PCR. Line 436 - 452 explain in details the occurrence of the other way around as well

35. **Study Limitations- Mention about the potential limitations of applying the molecular evaluation techniques like qPCR in actual community settings with the cost and resource limitations.**

Agreed, this has been added in the limitations section (Line 459 - 461)

36. **Conclusion- This wasn’t the case always. Refer to the comments above.**

Noted. However, overally the assay is sensitive compared as per the findings of the current study. Line 22

37. **References - Adhere to standard nomenclature principles in the appearance of scientific names**

The references have been revised and updated

Authors wish the version will satisfy the editor and reviewers and meet all standards of PLOS ONE. The authors welcome further comments, if any. We thank you too for your support.
